# Neuroprotective effect of *Bouvardia ternifolia* (Cav.) Schltdl via inhibition of TLR4/NF-κB, caspase-3/Bax/Bcl-2 pathways in ischemia/reperfusion injury in rats

**DOI:** 10.3389/fphar.2024.1471542

**Published:** 2024-09-23

**Authors:** Yury Maritza Zapata-Lopera, Gabriela Trejo-Tapia, Edgar Cano-Europa, Aida Araceli Rodríguez-Hernández, Placido Rojas-Franco, Maribel Herrera-Ruiz, Enrique Jiménez-Ferrer

**Affiliations:** ^1^ Centro de investigación Biomédica del Sur, Instituto Mexicano del Seguro Social, Xochitepec, Morelos, Mexico; ^2^ Centro de Desarrollo de Productos Bióticos, Instituto Politécnico Nacional, Yautepec, Morelos, Mexico; ^3^ Laboratorio de Metabolismo I, Departamento de Fisiología, Escuela Nacional de Ciencias Biológicas, Instituto Politécnico Nacional, Ciudad de México, Mexico; ^4^ CONAHCYT - Instituto Politécnico Nacional, Centro de Desarrollo de Productos Bióticos, Yautepec, Morelos, Mexico

**Keywords:** oxidant stress, cerebral ischemia, glial activation, inducible nitric oxide synthase, neuronal nitric oxide

## Abstract

**Introduction:**

*Bouvardia ternifolia* is a plant known for its traditional medicinal uses, particularly in treating inflammation and oxidative stress. Recent studies have explored its potential in neuroprotection, especially in the context of cerebral ischemia/reperfusion injury, a condition where blood supply returns to the brain after a period of ischemia, leading to oxidative stress and inflammation. This damage is a major contributor to neuronal death and neurodegenerative diseases.

**Methods:**

A BCCAO/reperfusion model was induced, followed by treatment with *B. ternifolia* extract. Various molecular biology methods were employed, including Western blot analysis, gene expression assessment via RT-qPCR, and the measurement of oxidative stress mediators.

**Results:**

In the BCCAO/reperfusion model, the compounds in the dichloromethane extract work by targeting various signaling pathways. They prevent the activation of iNOS and nNOS, reducing harmful reactive oxygen and nitrogen species, and boosting antioxidant enzymes like catalase and superoxide dismutase. This lowers oxidative stress and decreases the expression of proteins and genes linked to cell death, such as Bax, Bcl-2, and caspase-3. The extract also blocks the TLR4 receptor, preventing NF-κB from triggering inflammation. Additionally, it reduces the activation of microglia and astrocytes, as shown by lower levels of glial activation genes like GFAP and AiF1.

**Conclusion:**

The dichloromethane extract of *B. ternifolia* demonstrated significant neuroprotective effects in the BCCAO/reperfusion model by modulating multiple signaling pathways. It effectively reduced oxidative stress, inhibited inflammation, and attenuated apoptosis, primarily through the downregulation of key proteins and genes associated with these processes. These findings suggest that the extract holds therapeutic potential for mitigating ischemia/reperfusion-induced neuronal damage.

## 1 Introduction

The root and rhizome of *Rubia yunnanensis* Diels (a plant of the Rubiaceae family) is an indigenous medicine endemic to Yunnan, China; has pharmacological effects, such as antioxidant actions, to treat brain diseases such as tides and anemia caused by cerebral ischemia and antithrombosis (Cheng et al., 2024). The compounds reported for this plant are of the naphthoquinone type: blondequinones A–C, blonderbonone A, inhibit the protein phosphorylation of MAPKs (p38, ERK and JNK) under conditions of oxidative stress (Suyama et al., 2017); triterpenes: blonderbonol A and B promoted the activation of ERK1/2 and JNK pathway in MAPK family, which in turn increased the expression of p53; mediated JNK activation downregulated the expression of the anti-apoptotic protein Bcl-2. In addition, triterpenes were also found to inhibit the activation of NF-κB signaling by down-regulating the expression and attenuating the translocation to nucleus of NF-κB p65 (Liou and Wu, 2002); cyclic hexapeptides Rubiyunnanins A, B, C-H, inhibit nitric oxide (NO) production (Fan et al., 2010a) and bicyclic hexapeptidic glucoside: RA-XII has antitumor activity independent of apoptosis, and suppressed protective autophagy by regulating mTOR and NF-κB pathways (Wang et al., 2020). This application suggests that plants belonging to the Rubiaceae family such as *Bouvardia ternifolia* may have the potential to treat ischemic stroke*.* This plant is a shrub found in Mexico and Central America. It is commonly known as “trumpet” due to the shape of its flowers. It is also referred by the Nahuatl name “*ezpahtli*,” as documented in the De la Cruz-Badiano Codex. Traditionally, the root has been used to address heat and heart exhaustion, stop excessive bleeding, treat old sores, and relieve scorpion stings (Unam, 2024).

Previously, it was reported that both aqueous and ethyl acetate fractions and certain purified compounds such as bouvardin, scopoletin, and a new molecule ternifoliol derived from the root of *B. ternifolia* exhibit immunomodulatory effects. *In vitro* and *in vivo* models have demonstrated that these fractions decreased the synthesis of proinflammatory interleukins (IL-6, TNF-α, and IL-1β) and modulated the anti-inflammatory interleukins as IL-10 and IL-17 in joints and kidneys while also downregulated the expression of NF-κB and AP-1. In another study, Zapata-Lopera et al. (2023) reported the effect of dichloromethane and hexane extracts from the root of *B. ternifolia* in a neuroinflammation model induced with LPS (Zapata-Lopera et al., 2023).

Stroke (CVA) is a serious neurological condition characterized by the interruption of blood flow to the brain, resulting in tissue damage and impaired brain function. The causes of stroke can be mainly classified into two types: ischemic and hemorrhagic. The ischemic stroke accounts for approximately 85% of all strokes and occurs when a cerebral artery is obstructed, usually by a thrombus or embolus that blocks blood flow. The leading causes include atherosclerosis, where fatty plaques build up in arterial walls, and heart conditions like atrial fibrillation, which can lead to clot formation that travels to the brain. Hemorrhagic stroke constitutes around 15% of cases and occurs when a blood vessel in the brain ruptures, causing bleeding within or around the brain. The primary causes include chronic hypertension, which weakens blood vessel walls, and arteriovenous malformations, which are abnormal connections between arteries and veins that can rupture (Chamorro et al., 2016). Brain blood supply is essential for neurological functionality (Chamorro et al., 2016). Therefore, the interruption of cerebral blood flow, secondary to ischemic stroke, is one of the cerebrovascular diseases that cause a decrease in the supply of oxygen and glucose that sustain cellular homeostasis (Attwell et al., 2010). Interrupting blood flow causes excessive reactive oxygen species (ROS) generation. Eventually, it leads to selective death or loss of neurons, associated with behavioral-cognitive dysfunctions and/or sensorimotor alterations in experimental animal models (Venkat et al., 2017). Another consequence of hypoxia derived from the event of cerebral ischemia affects the oxidative defense mechanism of the brain since there is evidence that cerebral ischemia-reperfusion produces an imbalance between the cellular levels of prooxidants (e.g., superoxide, hydroxyl radical, hydrogen peroxide and peroxynitrite radicals) and antioxidant capacity (e.g., enzymatic and non-enzymatic systems) causing organic damage both in the vasculature and causes a decrease in cellular functions and leads to neuronal loss (Furian et al., 2024).

However, in the context of ischemia/reperfusion syndrome, a modification in pH, calcium levels, and the presence of oxygen-free radicals can compromise the integrity of the inner mitochondrial membrane, which leads to the depolarization of membrane potentials, cellular edema, and the release of pro-apoptotic molecules such as cytochrome c (Orellana-Urzúa et al., 2023). These processes can generate edema in the cytosol and, finally, activate genes related to cell apoptosis (Panconesi et al., 2022). Therefore, the primary factors contributing to reperfusion injury include oxidant stress, leukocyte infiltration, mitochondrial mechanisms, platelet activation and aggregation, complement activation, and blood-brain barrier disruption. These factors ultimately lead to brain edema or hemorrhagic transformation, causing significant neuronal death and neurological dysfunctions (Jiménez-Castro et al., 2019). Therefore, regulating the production of reactive oxygen species (ROS), proinflammatory cytokines (TNF-α, IL-1β, and IL-6), the release of pro-apoptotic molecules, and Toll-like receptor (TLR) signaling through the NF-κB pathway could be a promising strategy for treating ischemic stroke.

The main treatment for an ischemic stroke, or brain infarction, is a tissue plasminogen activator (tPA). This medication dissolves blood clots that block blood flow to the brain. After stroke, antiplatelet drugs (acetylsalicylic acid (aspirin) or clopidogrel) help prevent blood from clotting. Acetylsalicylic acid inhibits cyclooxygenase 1 and 2 (COX-1 and COX-2) irreversibly, interfering with the synthesis of prostaglandins, thromboxanes and prostacyclin. Clopidogrel selectively inhibits the binding of adenosine diphosphate (ADP) to its platelet receptor P2Y12 and the subsequent activation of the glycoprotein IIb-IIIa complex mediated by ADP, thus inhibiting platelet aggregation (Saidi and Kenari, 2014).

This work aimed to evaluate the neuroprotective effects of dichloromethane extract of *B. ternifolia*, using the *in vivo* cerebral ischemic stroke Bilateral Common Carotid Artery Occlusion (BCCAO)/reperfusion model, and the underlying genetical and molecular mechanisms.

### 1.1 The effect of *B. ternifolia* extract on oxidative stress markers

**FIGURE 1:**
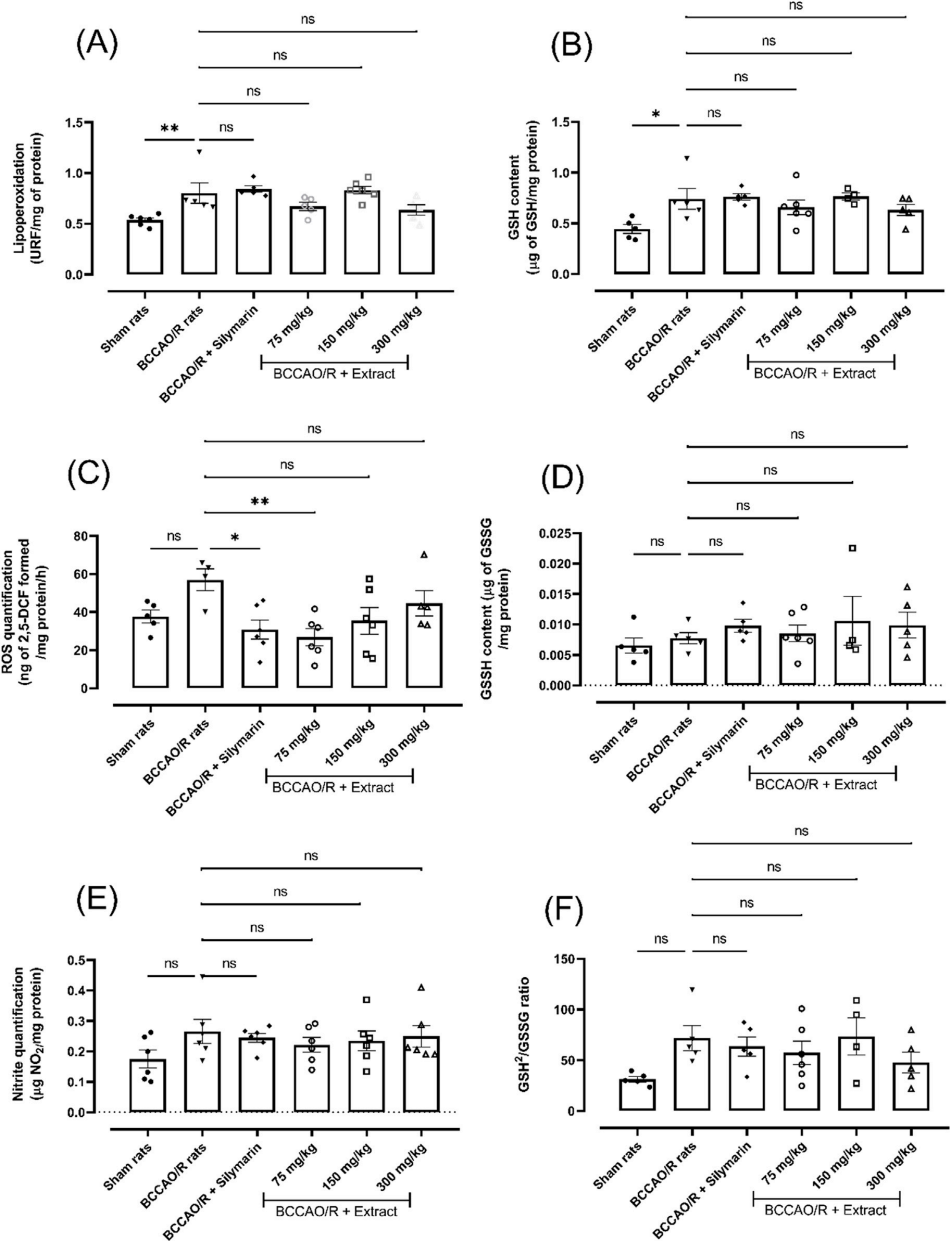
The effect of *B. ternifolia* extract at 75, 150, and 300 mg/kg on BCCAO/R-induced oxidative stress and alterations in the brain redox environment. Oxidative stress markers are presented in panels **(A, C, E)**. In contrast, redox environment markers are shown in panels **(B, D, F)**. RFU (relative fluorescence units) was used for quantification. Data are expressed as the mean ± SEM. The variables were assessed through a one-way analysis of variance (ANOVA) and the Dunnett *post hoc* test. (*) *p* < 0.05 (**) *p* < 0.01 vs. the BCCAO/R group. No significant difference (ns).

Reactive oxygen and nitrogen species can attack cell membranes, causing the oxidation of the fatty acids (LPO) that compose them and thereby causing the loss of the properties inherent to cell membranes. In order to evaluate the generation of reactive species, oxidative stress markers were evaluated after a BCCAO injury/brain reperfusion process, the *B. ternifolia* extract at different doses (75 mg/kg, 150 mg/kg, and 300 mg/kg) was orally administered. The main changes were showed on decreases in the production of ROS (∼54%, dose 75 mg/kg), LPO (∼22%, dose 300 mg/kg) and nitrites (∼16%, dose 75 mg/kg) (Figures 1A–C, respectively). The changes on reduction of glutathione and oxidized glutathione levels are presented in panels Figures 1D, E, respectively. Panel Figure 1F shows the GSH^2^/GSSG ratio, where a change in the redox environment is observed. The data suggest a reduction of ROS production after administration of *B. ternifolia* extract.

### 1.2 *B. ternifolia* extract reduces the expression level of *NF-κB* gene associated with inflammation

**FIGURE 2:**
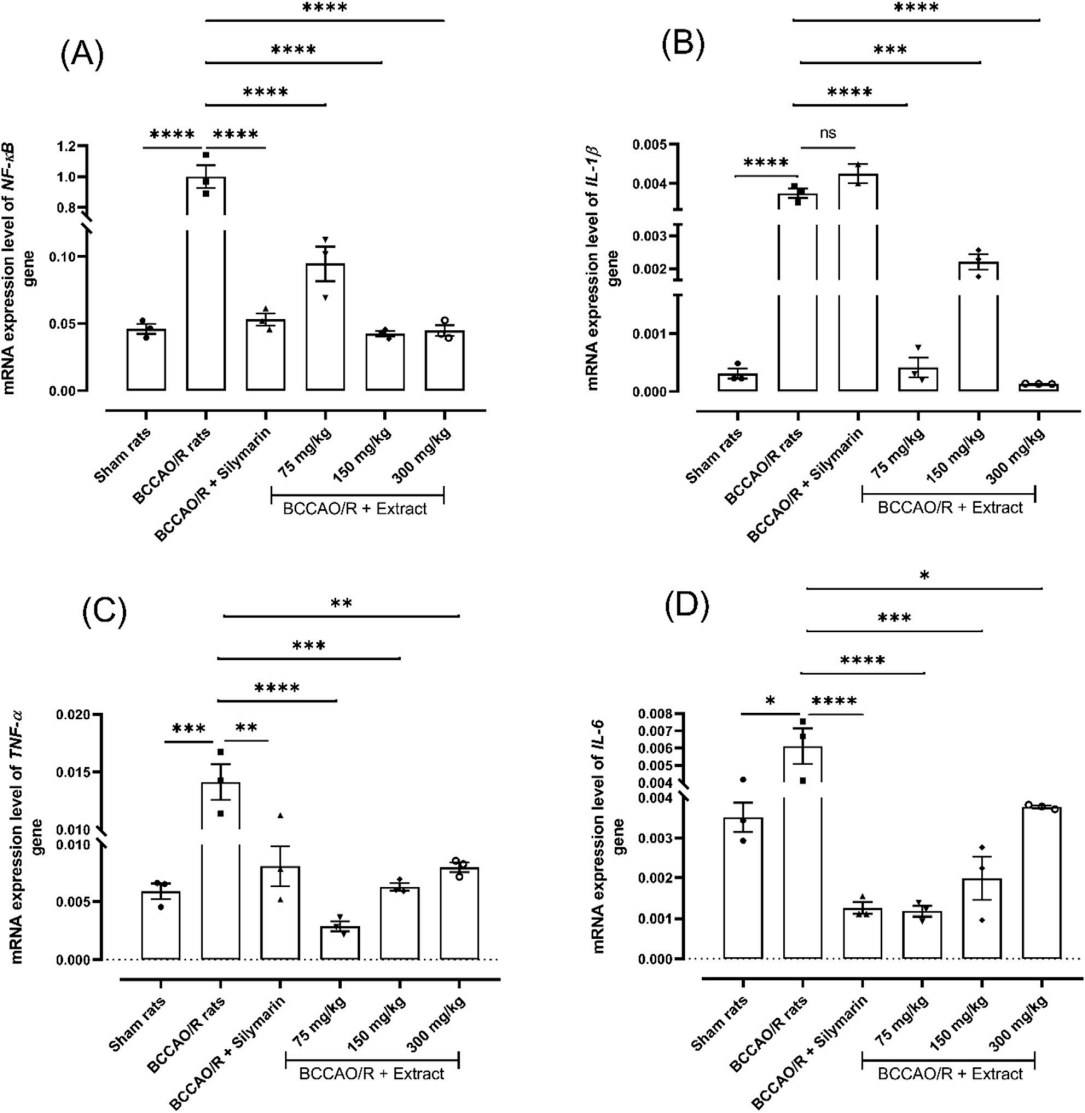
Effect of *B. ternifolia* extract at doses of 75, 150, and 300 mg/kg on the expression of genes associated with inflammation through the NF-κB pathway in the brain induced by I/R (BCCAO/R). Gene expression was evaluated for NF-κB **(A)**, IL-1β **(B)**, TNF-α **(C)**, IL-6 **(D)**. Data are expressed as the mean ± SEM. The variables were evaluated using a one-way analysis of variance (ANOVA) and a Dunnett *post hoc* test. (*) *p* < 0.05 (**) *p* < 0.01, (***) *p* < 0.001 (****) *p* < 0.0001, vs. BCCAO/R group. No significant difference (ns).

NF-κB is a crucial nuclear transcription factor that regulates the expression of many cytokines, including those that promote inflammation. The *B. ternifolia* extract was tested at different dose (75 mg/kg, 150 mg/kg, and 300 mg/kg), orally administered. A significant outcome of the study demonstrates that *B. ternifolia* extract markedly reduces the expression level of *NF-κB* gene with 0.094-fold for 75 mg/kg, 0.042-fold for 150 mg/kg, and 0.044-fold with 300 mg/kg in comparison with 1.0- fold in BCCAO/R treatment. Furthermore, there is a substantial decrease in the expression level of inflammatory interleukins genes such as *IL-1β* with dose 75 mg/kg and 300 mg/kg showing a 0.000414-fold and 0.000132-fold, respectively in comparison with 0.00375-fold in BCCAO/R treatment. The *TNF-α* and *IL-6* gene expression level gave the same behavior at 75 mg/kg, 0.0028-fold and 0.0011-fold, respectively compared to BCCAO/reperfusion rats. These findings suggest that *B. ternifolia* extract exhibits potential inhibitory effects on the NF-κB signaling pathway, which could confer protection against neuroinflammation and neurodegeneration (Figures 2A–D, respectively).

### 1.3 *B. ternifolia* extract influences a reduction in expression level of markers associated with neuronal apoptosis

**FIGURE 3:**
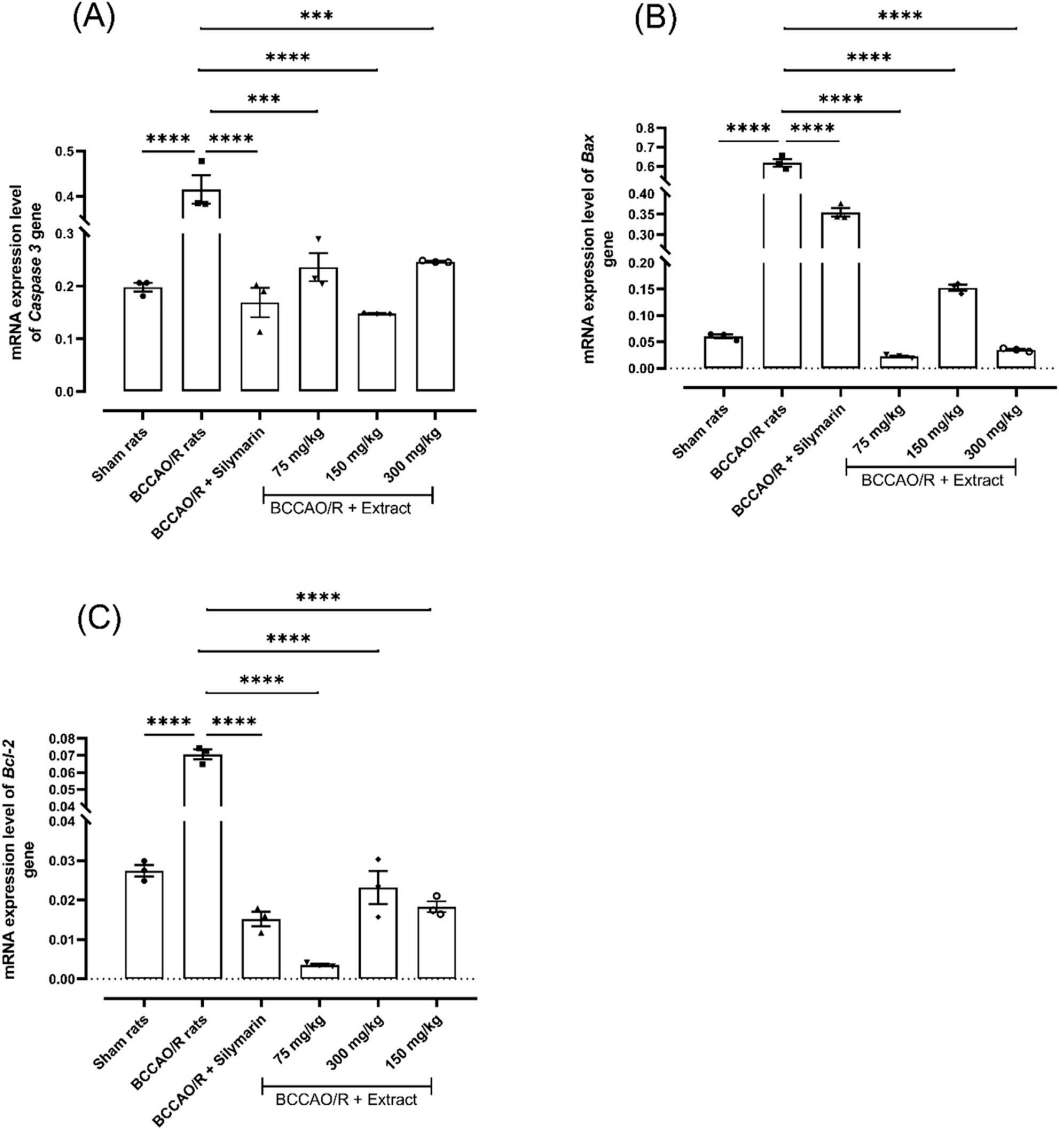
Effect of *B. ternifolia* extract at doses of 75, 150, and 300 mg/kg on the expression of genes associated with the apoptosis pathway in the brain induced by I/R (BCCAO/R). The expression of genes for caspase-3 **(A)**, BAX **(B),** and Bcl-2 **(C)** was evaluated. Data are presented as the mean ± SEM. The variables were assessed through a one-way analysis of variance (ANOVA) and the Dunnett *post hoc* test. (*) *p* < 0.001 (**) *p* < 0.0001 vs. the BCCAO/R group. No significant difference (ns).

To investigate the effects of the extract on cell damage following BCCAO/reperfusion, the expression level of genes associated with the apoptosis pathway was measured. As depicted in Figure 3, with a dose of 75 mg/kg, the *Bcl-2* gene showed a down expression level 0.0035-fold respect to 0.070-fold in BCCAO/reperfusion group, like profile expression level were found for *Bax* and *caspase-3* gene expression with 0.022-fold and 0.23-fold with a dose of 75 mg/kg compared to BCCAO (Figure 3).

### 1.4 *B. ternifolia* extract decreases the inflammatory mediators

**FIGURE 4:**
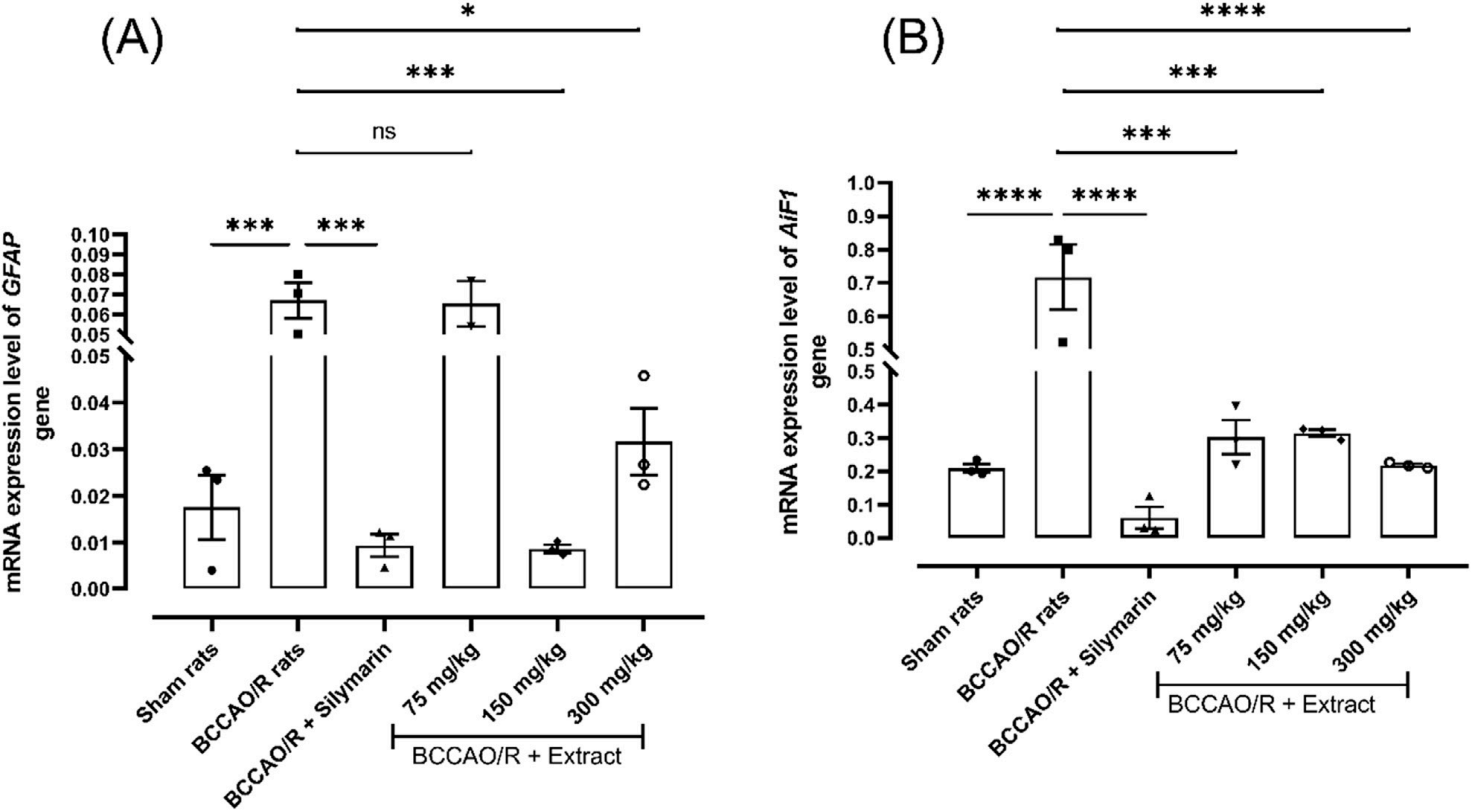
Effect of *B. ternifolia* extract at doses of 75, 150, and 300 mg/kg on the expression of genes associated with glial activation proteins in the brain induced by I/R (BCCAO/R) was examined. Gene expression was assessed for GFAP **(A)** and AiF1 **(B)**. Data are expressed as the mean ± SEM. The variables were analyzed using a one-way analysis of variance (ANOVA) and the Dunnett *post hoc* test. (*) *p* < 0.05 (**) *p* < 0.001, (****) *p* < 0.0001 vs. the BCCAO/R group. No significant difference (ns).

Astrocytes and microglia play a vital role in normal neuronal function and tissue repair after brain injury. The expression level of the *GFAP* and *AiF1* genes are a commonly used marker to measure astrocytic and inflammatory response in the brain. Figure 4 illustrates changes in the expression of genes associated with the activation of cells participating in the inflammatory process within the brain tissue, specifically microglia *AiF1* gene and astrocytes *GFAP* gene. Treatments with silymarin and the extract at 150 and 300 mg/kg led to a reduction in the expression level of the *GFAP* gene 0.0087- fold and 0.031-fold compared to 0.066-fold in BCCAO/R group (Figure 4A). Treatments with the extract at different doses and silymarin resulted in a decrease in the expression level of the *AiF1* gene 0.71-fold respect to BCCAO/R (Figure 4B). Notably, we found that the expression level was maintained in *AiF1* gene 0.30-fold at 75 mg/kg, 0.31-fold at 150 mg/kg and 0.21-fold at 300 mg/kg of the extract. These results prove that *B. ternifolia* extract decreases the production of inflammatory mediators such as the involved in glial activation, and cellular damage in rats after cerebral ischemic stroke.

### 1.5 Effect of *B. ternifolia* extract on iNOS, nNOS, TLR4, NF-κB and COX-2 proteins

**FIGURE 5:**
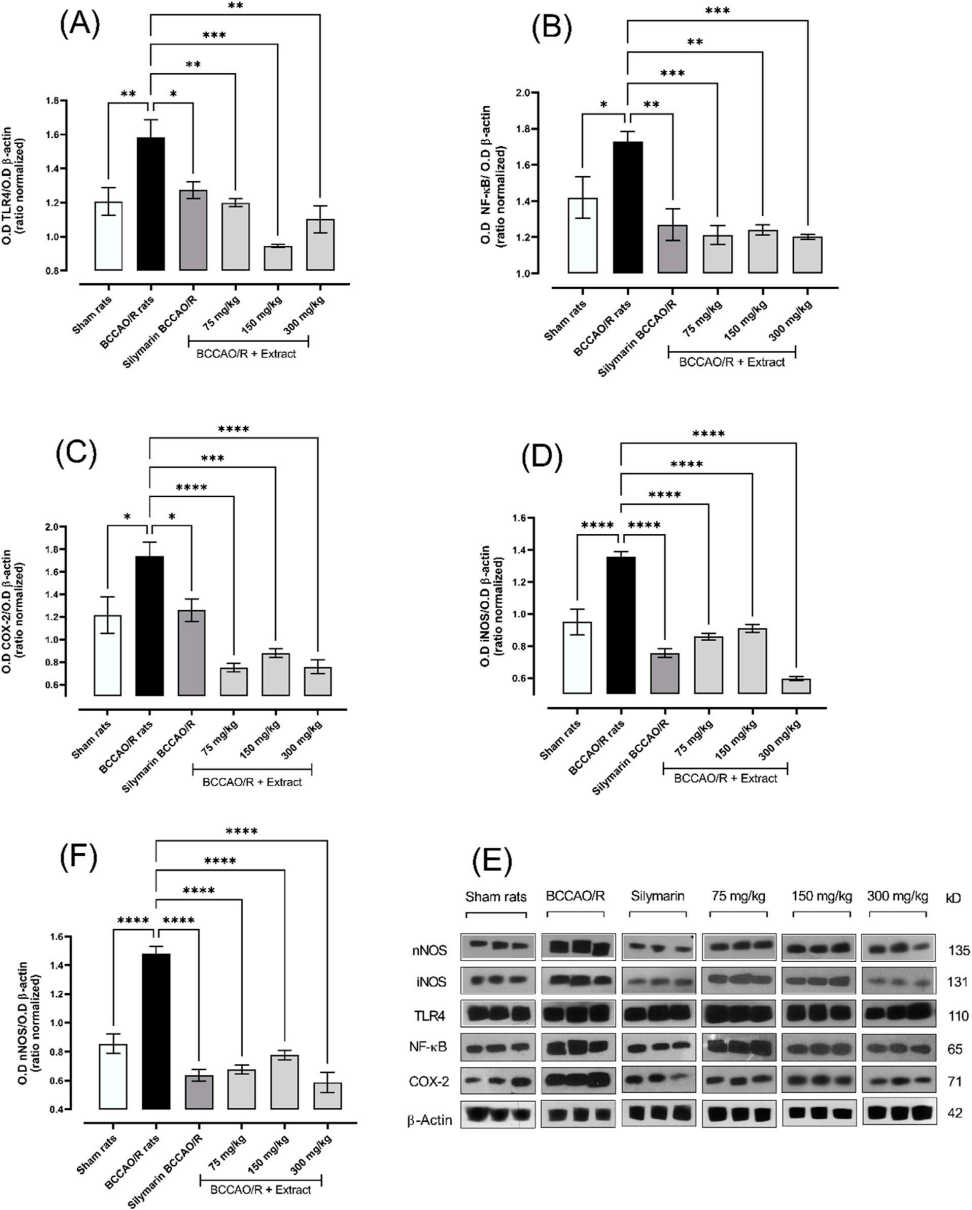
The effect of dichloromethane extract at 75, 150, and 300 mg/kg doses on I/R (BCCO/R) was investigated. Protein expression for TLR4 **(A)**, NF-κB **(B)**, COX-2 **(C)**, iNOS **(D)**, and nNOS **(F)** was assessed. Optical density (O.D) was measured. Additionally, a representative Western blot demonstrated the impact of dichloromethane extract on BCCO/R-induced brain stress via the NF-κB pathway **(E)**. Data are expressed as the mean ± SEM. The variables were analyzed using a one-way analysis of variance (ANOVA) and the Dunnett *post hoc* test. (*) *p* < 0.05 (**) *p* < 0.01, (***) *p* < 0.001 (****) *p* < 0.0001, vs. BCCAO/R group. No significant difference (ns).

Toll-like receptor 4 (TLR4) constitutes a transmembrane protein, which is expressed in microglia, astrocytes and neurons, and initiates pro-inflammatory cascades. The main receptors and mediators of this pathway are manifested during the ischemic process. The expression of the TLR4 protein (∼41%) exhibited a downregulation with the treatments used (75, 150, and 300 mg/kg), leading to a reduction in the expression of the NF-κB protein (∼70%) (Figures 5A–E). inducible nitric oxide synthetase (iNOS) and neuronal nitric oxide (nNOS), in response to cytokine production, react with O^−^, producing excess peroxynitrite and, consequently, cellular toxicity. Mitochondrial damage is evidenced by a decrease in the respiratory rate and an increase in the enzyme cytochrome-C oxidase, which favors the overproduction of superoxides. In Figures 5C, D–E, a decrease in the expression of the proteins iNOS (∼57%) and cyclooxygenase-2 (COX-2) (∼57%) is evident with each of the treatments. Neuronal nitric oxide synthetase (nNOS) (∼61%) also showed a decrease in expression with each of the treatments (Figures 5E, F).

### 1.6 Effect of *B. ternifolia* extract on BAX and Bcl-2 proteins

**FIGURE 6:**
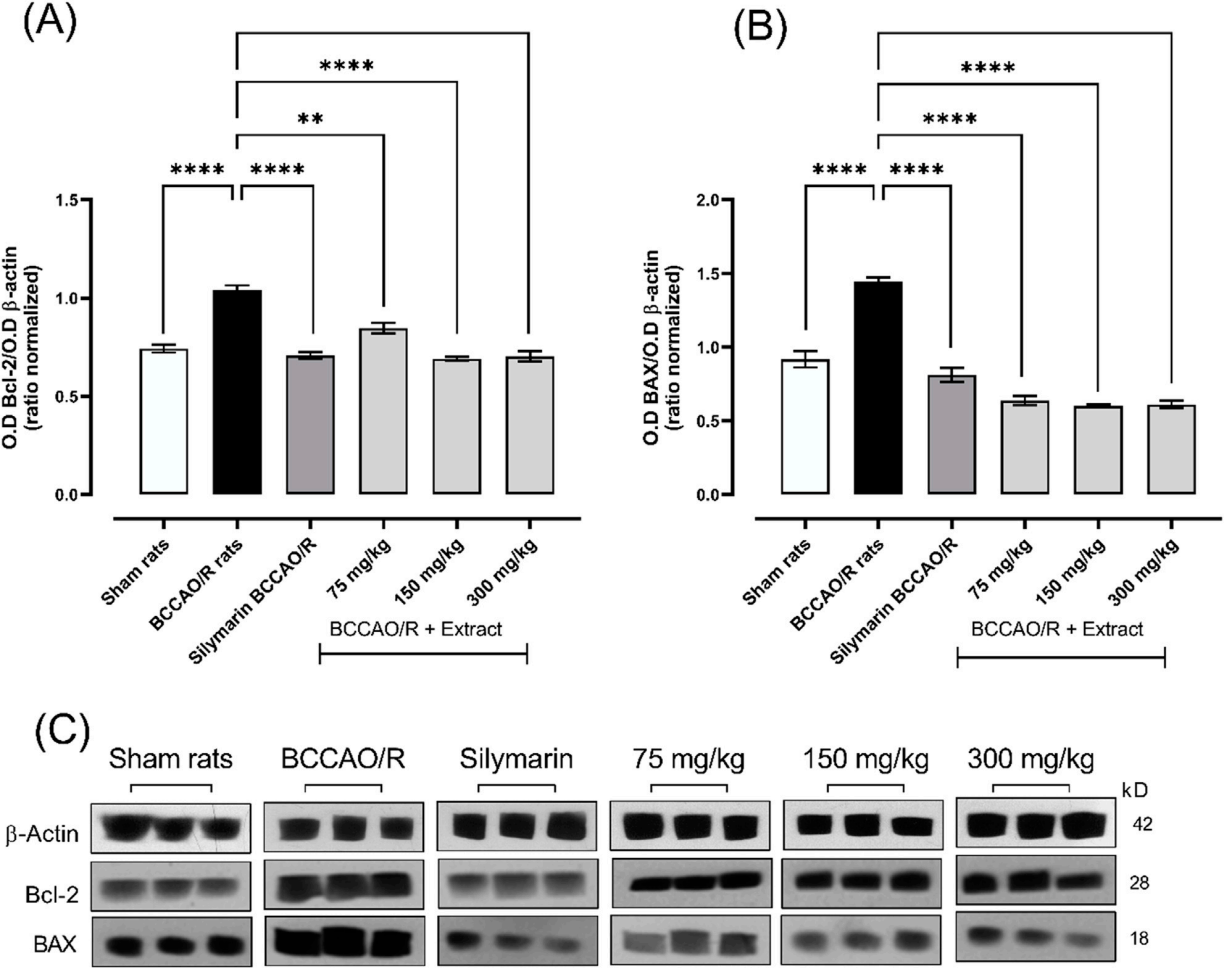
The effect of dichloromethane extract at 75, 150, and 300 mg/kg doses on I/R (BCCO/R) protein expression was investigated. Protein expression for Bcl-2 **(A)**, BAX **(B)**. Optical density (O.D) was measured. Additionally, a representative Western blot demonstrated the impact of dichloromethane extract on BCCO/R-induced brain stress via the BAX and Bcl-2 pathway **(C)**. Data are expressed as the mean ± SEM. The variables were analyzed using a one-way analysis of variance (ANOVA) and the Dunnett *post hoc* test. (*) *p* < 0.05 (**) *p* < 0.01, (***) *p* < 0.001 (****) *p* < 0.0001, vs. BCCAO/R group. No significant difference (ns).

In the context of apoptosis regulation in an experimental ischemia/reperfusion model, an increase in the anti-apoptotic protein Bcl-2 was observed in the BCCAO/R damage group, with a mean of ±1.03. The applied treatments, including silymarin and different doses of *B. ternifolia* extract, showed a Bcl-2 ratio like that of the healthy control group. Specifically, treatment with silymarin (Mean ± 0.70) and extract doses of 75 mg/kg (Mean ± 0.84), 150 mg/kg (Mean ± 0.69), and 300 mg/kg (Mean ± 0.70) did not significantly alter the Bcl-2 ratio compared to the healthy control, as illustrated in Figures 6A, C.

In contrast, the pro-apoptotic protein BAX showed an increase in the BCCAO/R group, with a mean of ±1.44, suggesting an increased predisposition to apoptosis. However, the treatments reduced the concentration of BAX compared to the injury group. Specifically, silymarin treatment reduced the mean BAX to ±0.81, while extract doses of 75 mg/kg, 150 mg/kg, and 300 mg/kg lowered the means to ±0.63, ±0.60, and ±0.61, respectively. These results, presented in Figures 6B, C, indicate that the treatments not only stabilize the Bcl-2 ratio but also contribute to the reduction of BAX, which may be associated with a lower degree of apoptosis in the ischemia/reperfusion model.

## 2 Discussion

In this research, it was shown that *B. ternifolia* extract has activity on different markers of ischemia/reperfusion cascade which is modeled using the bilateral common carotid artery occlusion (BCCAO/R) tool in rats, that is widely employed to simulate cerebral ischemia, providing insights into underlying mechanisms and potential therapeutic interventions for vascular brain disorders (Bansal et al., 2013). This experimental strategy involves ligating both common carotid arteries, resulting in reduced blood flow to the brain, causing diminished oxygenation and nutrition. Consequently, this can lead to neuronal death and cognitive dysfunction. Reperfusion following BCCAO affects various brain regions, including the cerebral cortex, hippocampus, and basal ganglia. Damage to these regions can impact cognitive and motor functions, emphasizing the model’s relevance (Wahul et al., 2018).

The BCCAO/R model employed in this study revealed an alteration in the REDOX environment, characterized by increased production of reactive oxygen species (ROS), lipoperoxidation, and nitrites. Under physiological conditions, ROS act as signaling molecules that regulate the contraction, relaxation, and growth of vascular smooth muscle cells. However, the balance between ROS (oxidants) and antioxidants can be disrupted in pathophysiological situations, leading to endothelial dysfunction. ROS can directly damage DNA, proteins, and lipids when present in excess, thereby contributing to cell death, necrosis, and apoptosis (Polopalli et al., 2022). The results from this study reveal an increase in ROS and LPO levels in the brain subjected to BCCAO/reperfusion injury. ROS and LPO are widely recognized as primary oxidative stress markers, and the dichloromethane extract notably mitigated their elevation. This data suggests that the chemical compounds in the extract possess antioxidant properties (Förstermann and Münzel, 2006).

The production of reactive oxygen species (ROS) can induce cellular damage, encompassing mitochondrial dysfunction, disruption of the cell membrane, and increased cell permeability (Habtemariam, 2019). Conversely, antioxidants, present in lower concentrations than the oxidizable substrate, play a crucial role in significantly reducing or preventing the harmful effects of reactive species (Shields et al., 2021). Moreover, oxidative damage may deplete intracellular antioxidants (such as GSH and SOD), leading to the production of malondialdehyde (MDA). Studies have reported that brain BCCAO/R injury could escalate ROS generation, inducing oxidative stress by suppressing the activities of antioxidant biomarkers.

The observed reduction in nuclear translocation of NF-κB p65 and the decrease in IκB phosphorylation upon treatment with the dichloromethane extract from *B. ternifolia* suggest that the extract may exert its anti-inflammatory effects by modulating the NF-κB signaling pathway. By interfering with NF-κB activation, the extract could help mitigate the expression of inflammatory cytokines and protect against neuroinflammation and associated neurodegenerative processes. This further supports the potential neuroprotective properties of *B. ternifolia* extract in the context of cerebral ischemia/reperfusion injury.

Indeed, in eukaryotic cells, the NF-κB protein is a vital nuclear transcription factor with a significant role in regulating the expression of numerous cytokines, including those involved in promoting inflammation. In various diseases, NF-κB has been recognized as a potential target for inhibiting the inflammatory response. Accumulating evidence supports the notion that overexpression of NF-κB contributes to brain injury induced by ischemia. Targeting NF-κB may represent a therapeutic approach to mitigate inflammation associated with cerebral ischemia/reperfusion injury (Shal et al., 2018).

The significant decrease in TLR4 expression with the administration of *B. ternifolia* dichloromethane extract in this study suggests that the extract may modulate the TLR4-mediated signaling pathway. By inhibiting TLR4 expression, the extract could interfere with initiating downstream inflammatory cascades, including the NF-κB pathway. Also, may contribute to the observed anti-inflammatory effects of dichloromethane extract in the context of cerebral ischemia/reperfusion injury.

The Toll-like receptor 4 (TLR4) is indeed a crucial component of the innate immune system. In the context of cerebral ischemia/reperfusion injury, TLR4 has been implicated in the inflammatory response. The observed increase in TLR4 expression in rats subjected to BCCAO/R injury aligns with the idea that TLR4 contributes to the BCCAO/R-induced inflammatory response, as mentioned in the Dong *et al.* study (Jover-Mengual et al., 2021).

In this research, it was observed that the dichloromethane extract administered at different doses attenuated the expression level of the genes associated with *IL-1β*, *IL-6*, *COX-2*, and *TNF-α* compared to the BCCAO/reperfusion group. In response to induced cerebral BCCAO/reperfusion, there was an upregulation of genes associated with proinflammatory cytokines in the brain. These cytokines, secreted by, glial cells, and neurons, play a crucial role in the inflammatory response. Notably, IL-1β, IL-6, COX-2, and TNF-α emerged as key contributors to brain inflammation during ischemic injury, with IL-1β and TNF-α potentially intensifying the extent of brain damage (Dong et al., 2021).

Microglia, resembling resident macrophages in the brain, play a pivotal role in maintaining homeostasis and defending and repairing host cells. Conversely, astrocytes provide structural, metabolic, and trophic support to neurons while acting as immunocompetent cells capable of secreting inflammatory mediators. However, chronic activation of both cell types can result in the excessive secretion of proinflammatory molecules, including TNFα, IL-6, and IL-1β, potentially harming neuronal viability. Indeed, diseases such as Alzheimer’s and Parkinson’s have been associated with heightened microglial activation, astrogliosis, and increased expression of *TNFα*, *IL-1β*, and *IL-6* (Du et al., 2019). In this study, an increase in the activation of microglia was observed, determined by the expression marker *Iba-1*, and a significant reduction in their expression and activation was demonstrated with the dichloromethane extract. Astrocytes are crucial glial cells that play a vital role in normal neuronal function and tissue repair following brain injury. *GFAP* expression is commonly used as a marker to measure astrocytic activity and the inflammatory response in the brain (Castellano et al., 2019). Therefore, this study demonstrated a decrease in this protein with the administration of the extract after BCCAO/reperfusion injury.

Apoptosis is one of the most critical mechanisms in the pathophysiology of cerebral infarction. *Bcl-2* is a protein known for its ability to prevent apoptosis, as it binds to Bax and prevents the formation of a pore in the mitochondria that would cause the release of toxic substances. Upregulation of *Bax*, mediated by p53, and downregulation of *Bcl-2* could promote the release of cytochrome c from mitochondria and the activation of caspase-3, which triggers the apoptosis process (Deng et al., 2018). In this study, a significant decrease in the levels of apoptotic mediators, such as Bax and caspase 3, was observed after administration of different doses of dichloromethane extract compared to the damaged control group. Furthermore, a decrease in the levels of the pro-apoptotic mediator *Bcl-2* was observed in the group treated with the extract compared to the damaged control group. These results demonstrate that the use of *B. ternifolia* extract significantly reduced the expression of *Bax* and *caspase-3* and *Bcl-2* in rats with cerebral ischemia-induced for 60 min and reperfusion for 24 h.

The dichloromethane extract of the root of *B. ternifolia* in its chemical composition contains: α-Tocopherol, this has chromane ring with a hydroxyl (OH) group at position six and a phytyl side chain at position 2. This side chain comprises an isoprenoid hydrocarbon chain with three methyl groups (CH_3_) at position two and one ethyl group (CH_2_-CH_3_) at position 3. The antioxidant activity of vitamin E arises from its capacity to donate electrons, neutralizing free radicals unstable and highly reactive molecules capable of damaging cells and tissues. It is a fat-soluble antioxidant in cell membranes and safeguards them from oxidative damage (Lucken-Ardjomande and Martinou, 2005). It has also been reported to inhibit the production of pro-inflammatory cytokines (e.g., TNF-α) and anti-inflammatory (e.g., IL-10). Additionally, it affects the activation of receptors such as TRL4 and CD40, which modulate the PI3K-Akt signaling pathway (Hobson, 2016); the squalene possesses antioxidant properties, neutralizing free radicals and protecting cells from oxidative damage. The structure of squalene, characterized by multiple conjugated double bonds, contributes to its antioxidant and anti-inflammatory capacity (La Torre et al., 2023); 3-Carene, a terpene, has demonstrated, in various studies, its ability to reduce the production of inflammatory cytokines (Ibrahim and Naina Mohamed, 2021) and COX-2 (Kim et al., 2020). Another secondary metabolite found in the extract, lupeol, has been highlighted in research for its antioxidant properties and impact on modulating astrocyte morphology. Furthermore, it has been observed to induce the regulation of mRNA expression for pro-inflammatory markers such as TNF-α and iNOS, in addition to influencing the production of nitric oxide and IL-6 (Huang et al., 2019); ternifolial features a chemical structure composed of a pentasubstituted aromatic ring linked to a cyclopentane. The hydroxyl groups within this structure release hydrogen atoms, which, in turn, combine with free radicals, effectively blocking the chain reaction mediated by reactive oxygen species and thereby reducing oxidative stress. Furthermore, the double bonds in the structure can neutralize reactive oxygen species, manifesting their antioxidant action. Ternifolial also encompasses an aldehyde carbonyl group and an ester group, potentially influencing its pharmacological activity; The glycosylated cyclic hexapeptide in the extract has undergone prior investigation for its cytotoxic activity *in vitro* cultures of cancer cell lines. Additionally, its demonstrated ability to inhibit nitric oxide production in the LPS model and in RAW 264.7 murine macrophages induced by IFN-ϒ, along with its capacity to inhibit the activation of NF-κB and TNF-α, underscores its pharmacological potential (Oliveira-Junior et al., 2019).

**FIGURE 7:**
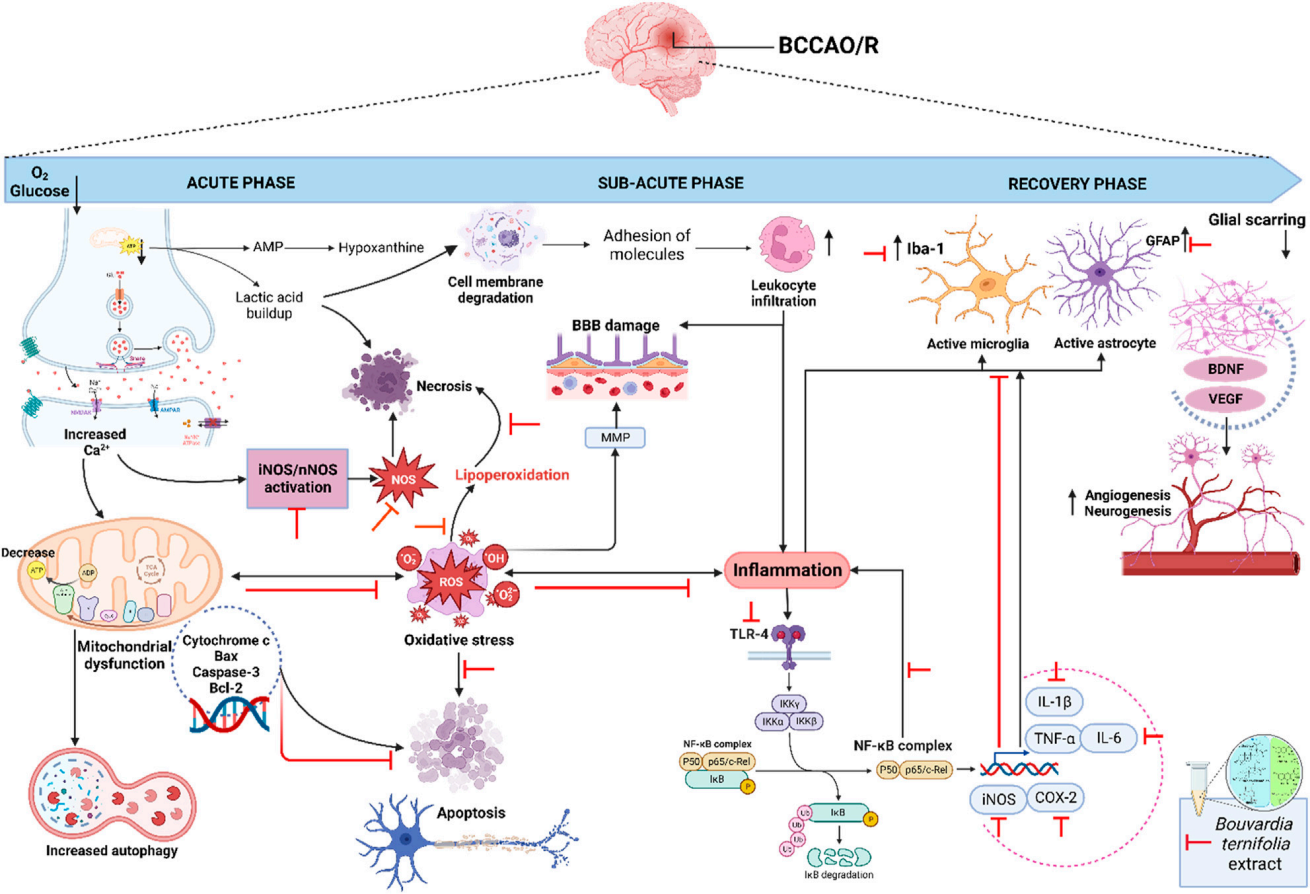
Proposed model for the protective of dichloromethane extract from *B. ternifolia* root on the different signaling pathways involved in the BCCAO/reperfusion model. After BCCAO/reperfusion there is a decrease in oxygen and glucose, there is an increase in the activation of glutamate receptors and an increase in intracellular calcium leading to the activation of inducible nitric oxide and neuronal nitric oxide that trigger the production of reactive nitrogen and oxygen species. The increase in calcium also generates dysfunction at the level mitochondrial and an increase in autophagy, as well as the release of proapoptotic cytochrome c mediators such as caspase 3 and Bax that intervene in apoptosis. On the other hand, the accumulation of lactic acid degrades cell membranes and causes necrosis, as well as lipoperoxidation through the oxidative stress produced. The process of Inflammation generates damage to the blood-brain barrier, infiltration of leukocytes and activation of the NF-κB pathway through its TLR4 receptor. Further downstream, the NF-κB complex translocate to the nucleus and activates inducible nitric oxide, COX-2, and the interleukins IL1-β, IL-6 and TNF-α, which in turn activate microglia and astrocytes through their membrane receptors. Several transcription factors are involved in the recovery phase at the genetic level, including brain-derived neurotropic factor (BDNF) and vascular endothelial growth (VEGF), both help angiogenesis and neurogenesis. Compounds present in crude dichloromethane extract attenuate several signaling pathways involved after ischemia/reperfusion injury. The red line represents the reduced or inhibits pathway. Created with Biorender.com.

In summary, Figure 7 shows the possible mechanism of dichloromethane extract in the BCCAO/reperfusion model. The extract modulates oxidative stress markers, TLR4/NF-κB/IL-1β/IL-6/TNF-α/COX-2 pathway, protein expression of iNOS, nNOS and genes expression *GFAP*, *Iba-1*, *caspase-3*, *Bcl-2*, and *Bax*.

## 3 Conclusion

The dichloromethane extract exhibits a neuroprotective effect, reducing neuronal death by mitigating oxidative stress markers. Analysis of the transcription level of the *NF-κB* gene that codify a transcription factor revealed that the dichloromethane extract from *B. ternifolia* downregulates its expression, indicating inhibition through the TLR4 receptor. This negative modulation extends to the expression of inflammatory mediators, including *IL-1β*, *IL-6*, and *TNF-α* genes. Furthermore, the administration of the extract resulted in a decrease in the expression of the genes, suggesting potential inhibition of both intrinsic and extrinsic pathways involved in programmed cell death of neurons. Additionally, genes associated with glial activation, such as, *GFAP* and *AIF1* genes exhibited decreased expression in the presence of the extract, possibly influencing the modulation of microglia and astrocyte activation during the early phase of BCCAO/reperfusion damage. Similarly, evaluation of protein expression associated with the activation pathway of the NF-κB transcription factor in the presence of the extract showed a pattern resembling that found in the transcripts, indicating inhibition of activation through the TLR4 receptor and negative modulation of protein expression, including iNOS, nNOS, and COX-2, after BCCAO/reperfusion damage (Figure 7). Under normal conditions, NF-κB associates with its inhibitory protein IκB and is retained in the cytoplasm. Upon activation, IκB kinases (IKKs) are activated and lead to phosphorylation of IκB, inducing its proteasome-mediated degradation and transport of NF-κB to the nucleus from the cytoplasm. Once in the nucleus, NF-κB binds to its binding sequence to activate relevant promoters and trigger the expression of inflammatory cytokines, such as IL-1β, IL-6, COX-2, and TNF-α. These facts indicate that NF-κB plays a critical role in regulating inflammation, and that inhibition of NF-κB may protect against neuroinflammation and neurodegeneration.

## 4 Materials and methods

### 4.1 Plant material and extract preparation

A sample of 1.7 kg of *B. ternifolia* root was gathered in Coajomulco, Municipality of Huitzilac, Morelos, México (Latitude 18.78, Longitude −99.23, direction 176.8), at an elevation of 1,124 m above sea level. A specimen has been archived in the Herbarium of the INAH Botanical Garden and authenticated by biologists Margarita Avilez and Macrina Fuentes, with the voucher number (INAH-MOR-2080). The root was processed, and the low polarity dichloromethane extract was obtained following the methodology already reported (Saidi and Kenari, 2014). The following compounds were reported in the dichloromethane extract: (1) 3-Carene, (2) 1H-Inden-1 one, 5-(1,1-dimethylethyl)-2,3-dihydro-3,3-dimethyl, (3) Lup-20 (29)-en-3-ol-acetate, (3β), (4) 2-Nonadecanone, (5) squalene, (6) α-Tocopherol, (7) s-indacene-1,7-dione, 2,3,5,6-tetrahydro-3,3,5,5-tetramethyl, (8) cyclic hexapeptide.

### 4.2 Animals for experimentation

All trials were conducted using 9-week-old male Sprague-Dawley rats, averaging a weight of 350 ± 50 g. The rats were individually housed in cages maintained at an ambient temperature of 25°C ± 1°C and humidity of 50% ± 10%, following a 12-h light/dark cycle, and were provided unrestricted access to food and water. The experiments strictly adhered to Mexican regulations governing experimental animal care (NOM062ZOO-1999). The protocol received approval from both the Institutional Research Committee and the Ethics Committee of the Mexican Institute of Social Security (IMSS) under registration number R-2020-1702-033, and it adhered to the ARRIVE guidelines (https://arriveguidelines.org).

### 4.3 Induction of cerebral ischemia by bilateral common carotid artery occlusion (BCCAO)

The rats were anesthetized using 10% Ketamine and 2% Xylazine. a) The rat was placed in the supine position and an incision was made in the midline of the neck. The submandibular glands, thyrohyoid muscles (TM), and sternocleidomastoid muscles (SM) are observed. b) The SM separate laterally. Once both ACC are exposed and dissected, they are ligated with vascular clips close to their division into external and internal carotid arteries. The ligation is maintained for 60 min.

The rats were divided into five groups (n = 7 per group): group I, healthy control with simulation of surgery (Sham), Group II, damage control, bilateral common carotid occlusion and reperfusion for 60 min (BCCAO); group III, BCCAO + treatment with silymarin at 50 mg/kg; group IV, BCCAO + treatment with dichloromethane extract at 75 mg/kg; group V, BCCAO + treatment with dichloromethane extract at 150 mg/kg; group VI, BCCAO + treatment with dichloromethane extract at 300 mg/kg.

### 4.4 Brain and sample preparation

All animals were euthanized 24 h after ischemia-reperfusion was induced using an overdose of sodium pentobarbital (100 mg/kg body weight, intraperitoneal), followed by intracardial administration of an isotonic sodium chloride solution. Subsequently, the brain was promptly extracted and frozen at −80°C.

### 4.5 Measurement of oxidative stress biomarkers

One hemisphere of the brain was homogenized in 2 mL of phosphate buffer (10 mM, pH 7.4) for subsequent biochemical determinations and Western blot analysis. The assessed oxidant stress markers included reactive oxygen species (ROS), lipid peroxidation, and nitrite quantification. Additionally, redox environmental markers, namely reduced glutathione (GSH), oxidized glutathione (GSSG), and their ratio (GSH^2^/GSSG), were evaluated using spectrophotometric techniques. The activity of antioxidant enzymes such as catalase, superoxide dismutase (SOD), Mn-SOD and Cu-Zn SOD was also evaluated as described above with some modifications (Fan J. T. et al., 2010).

#### 4.5.1 Determination of lipid peroxidation (LPO)

Lipid peroxidation was assessed by the formation of fat-soluble fluorescence, as previously described (Garcia-Pliego et al., 2021). In summary, each tissue sample was homogenized in 2 mL of phosphate solution (pH 7.4). Subsequently, 200 µL aliquots were combined with 4 mL of chloroform-methanol (2:1, v/v). Following a 15-s stirring period, the mixture was cooled on ice for 30 min to facilitate phase separation. The chloroform phase was then measured using a luminescence spectrophotometer (Perkin-Elmer LS-55; Canada) at excitation and emission wavelengths of 370 nm and 430 nm, respectively. The sensitivity of the spectrophotometer was set at 140 fluorescence units, employing 1 μg/mL quinine sulfate in 0.05 M H_2_SO_4_. Results were expressed as relative fluorescence units (RFU) per milligram of protein.

#### 4.5.2 Quantification of reactive oxygen species (ROS)

ROS were measured by formation of 2′7′-dichlorofluorescein (DCF) (Ali et al., 1992). Two microliters (2 μL) of brain homogenates were combined with 1,948 μL of TRIS-HEPES (18:1) and then incubated with 50 μL of dichlorofluorescein 2′7′-diacetate (DCFH-DA) for 1 h at 37°C. The reaction was halted by freezing. Fluorescence was measured on a luminescence spectrophotometer (Perkin-Elmer LS-55; Canada) at wavelengths of 488 nm (excitation) and 525 nm (emission). The results were expressed as pmol DCF formed/mg protein/h (Santamaría and Ríos, 1993).

#### 4.5.3 Determination of nitrites

Nitrites were evaluated by the Griess reaction, as previously described (Cano-Europa et al., 2008). Nitrites were evaluated as indirect markers of stress produced by reactive nitrogen species. A 100 μL portion of the homogenate was mixed with 500 μL of concentrated hydrochloric acid and 500 μL of 20% zinc suspension. The resulting mixture was shaken and incubated at 37°C for 1 h, followed by centrifugation at 4,000 × g for 2 min. Subsequently, the supernatant (50 μL) was added to a 96-well polystyrene plate containing 50 μL of 0.6% sulfanilamide and 0.12% N-(naphthyl)-ethylenediamine, and the plate was incubated for 15 min at room temperature. Absorbance was then measured at 530 nm using a Multiscan plate spectrophotometer (Go^®^ (Thermo Scientific™ Multiskan™ GO, Madrid).

#### 4.5.4 Evaluation of the antioxidant system

##### 4.5.4.1 Determination of reduced glutathione (GSH), oxidized glutathione (GSSG) and GSH^2^/GSSG index

To quantify the markers of the redox environment GSH and GSSG, the Hissin and Hilf procedure was used (Sastry et al., 2002). In summary, the brain was homogenized in 2 mL of 10 mM phosphate buffer, pH 7.4. Homogenates (150 μL) underwent treatment with 30% phosphoric acid and subsequent centrifugation at 10,000 g for 15 min. For GSH determination, 30 μL of a 1:10 diluted supernatant with FEDTA (100 mM phosphate and 5 mM EDTA) was mixed with 1.9 mL of FEDTA, followed by reaction with 100 µL of o-phthaldialdehyde. To assess GSSG, 75 μL of supernatant was combined with 35 μL of N-ethylmaleimide, and after 30 min, 60 μL of the mixture was homogenized in 1.84 mL of FEDTA. Subsequently, 100 μL of o-phthaldialdehyde was added, and fluorescence was measured using a luminescence spectrophotometer (Perkin-Elmer LS-55, Canadá) at excitation and emission wavelengths of 320 nm and 420 nm, respectively. The results were expressed as nmoles of GSH or GSSG per milligram of protein. The GSH^2^/GSSG ratio of the redox environment described by Schafer and Buettner was used as an indicator (Hissin and Hilf, 1976).

#### 4.5.5 Protein quantification

The protein concentration in the homogenates was measured by the Bradford method (Huang et al., 2000).

### 4.6 Quantitative real-time RT-PCR

qRT-PCR was performed with the StepOne Real-Time PCR Detection System and StepOne Software v2.1 (Applied Biosystems, Foster City, Ca, United States). The amplification reactions were performed in a total volume of 10 μL, the mixture contained 50 ng of total RNA treated as templated, 5 μL of iTaq universal SYBR Green reaction mix (2x) (Bio-Rad), 0.125 μL of iScript reverse transcriptase, 0.3 μL of the forward and reverse oligonucleotides. All samples were amplified in triplicate in a 48-well plate, with the following conditions: 10 min at 50°C (cDNA synthesis), 1 min at 95°C (polymerase activation) followed by 40 cycles at 95°C (DNA denaturation), 1 min at 58°C for gene *Bax*; 1 min at 60° for genes *β-actin*, *Bcl-2*, *caspase 3, NF-κB, AiF1;* 1 min at 55° for genes IL-1β, IL-6; 1 min at 58° for genes *TNF-α* and *Bax;* 1 min at 61° for gene *GFAP* (alignment/extension), RT-qPCR was used to quantify mRNA expression using the following primers: *Bax*, forward 5′- *ACA CCT GAG CTG ACC TTG GA*-3′, reverse 5′-*AGT TCA TCG CCA ATT CGC CT*-3’; *Bcl-2*, forward 5′- *CTG GTG GAC AAC ATC GCT CT*-3′, reverse 5′-*GCA TGC TGG GGC CAT ATA GT*-3; *caspasa-3*, forward 5′- *GGA GCT TGG AAC GCG AAG AA* -3′, reverse 5′- *ACA CAA GCC CAT TTC AGG GT* -3; *Iba-1* (*AiF1*), forward 5′- *TCT GAA TGG CAA TGG AGA TA*-3′, reverse 5′- *GTT GGC TTC TGG TGT TCT* -3; *NF-κB*, forward 5′- *TTC CCT GAA GTG GAG CTA GGA*-3′, reverse 5′- *CAT GTC GAG GAA GAC ACT GGA* -3; *IL-1β*, forward 5′- *GGG TCT GAC TCC CAT TTT CC* -3′, reverse 5′-*TCT GTG ACT CGT GGG ATG ATG AC*-3; *IL-6*, forward 5′-*CAG AGT CAT TCA GAG CAA TAC* -3, reverse 5′-*CTT TCA AGA TGA GTT GGA TGG*-3′ and *TNF-α,* forward 5′-*CCC CGA CTA TGT GCT CCT CAC*-3, reverse 5′- *AGG GCT CTT GAT GGC GGA* -3´ (Sigma-Aldrich, St. Louis, MO, United States). Each reaction was carried out according to the conditions found in the literature (Bradford, 1976; Mao et al., 2018; Arfuzir et al., 2020; Yardim et al., 2020; Shi et al., 2021). The expression of the housekeeping gene glyceraldehyde-3-phosphate dehydrogenase (*GAPD*) was also evaluated as an internal control forward 5′- *AGT GCC AGC CTC GTC TCA TA-3′*, reverse 5′- *GAT GGT GAT GGG TTT CCC GT* -3´ (Sigma-Aldrich, St. Louis, MO, United States). The Melt curve was carried out with cycles of 65°C–95°C for 15 s, increasing 0.5°C for each cycle. The relative levels of mRNA expression were determined by calculating the cycle threshold (Ct) values by calculating the difference between the Ct value of the gene evaluated–the control gene, expressed as ΔCt, and thereby applying it to the formula 2^−ΔCt^ (Livak and Schmittgen, 2001). Each sample was performed in triplicate (n = 3).

### 4.7 Western blot analysis TLR4, NF-κβ, COX-2, NOS-1, NOS-2, BAX and Bcl-2

Protein expression was determined with Western blot assays. Briefly, Samples were prepared by combining 50 μL of the homogenate with 5 μL of the protease inhibitor cocktail^®^ (MilliporeSigma, Burlington, MA, United States) in lysis buffer, followed by the addition of 45 μL of 2x Laemmli loading buffer (Biorad, Hercules, CA, United States, 161-0737). The mixture was homogenized by vortexing, subjected to a 3-min boiling water bath, and then stored at −20°C. A total of 50 μg of protein samples (3 μL) were loaded onto a 10% sodium dodecyl sulfate polyacrylamide gel (SDS-PAGE) and separated by electrophoresis (70 V for 120 min). Subsequently, proteins were electrotransferred from the gels to PVDF membranes in a Trans-Blot Turbo system (Biorad) at 25 V and 2.05 A for 12 min.

Following transfer, the membranes were blocked for 1 h with constant agitation in PBST (PBS with 0.05% Tween 20% and 5% low-fat milkSvelty^®^). This was followed by an overnight incubation at 4°C in blocking buffer containing primary antibodies. The primary antibodies (Santa Cruz Biotechnology, Dallas, TX, United States) were diluted 1:1,000 for NOS1 (sc-5302), NOS2 (sc-7271), COX-2 (sc-23983), TLR-4 (sc-293072), p-NF-κB-p65 (Ser 5361, sc-101752), BAX (2D2, sc-20067, #lot H1320), Bcl-2 (C-2, Sc-7382, lot# J2819), and were purchased from Byorbit (Cambridgeshire, Cambridge, United Kingdom) and diluted 1:1,000. After incubation, membranes were washed three times with fresh PBST (15 min/wash) and then incubated with a secondary antibody diluted 1:1,500 (HPR-conjugated goat anti-rabbit; Life Technologies, Rockford, IL, United States, 65-6120) and (HPR-conjugated goat anti-mouse; Life Technologies, Rockford, IL, United States, 65–6120) at room temperature for 1 h with constant stirring. The membranes were washed four times with fresh PBST.

Finally, protein bands were revealed on photographic plates (JUAMA, México City, México) using chemiluminescence and Luminata TM Forte^®^ (MilliporeSigma, Burlington, MA, United States). β-Actin protein expression served as the loading control and constitutive protein (Santa Cruz Biotechnology; sc-47778, 1:4,000 dilution). The optical density (OD) of all bands was quantified using ImageJ version 1.53t software (NIH, Bethesda, MD, United States) and expressed as the protein/β-actin ratio (Blas-Valdivia et al., 2021).

### 4.8 Statistical analysis

The data was analyzed using GraphPad^®^ Prism9 (GraphPad Software Inc., La Jolla, CA, United States). The data are presented as the mean ± standard error (SEM). The variables were assessed using one-way analysis of variance (one-way ANOVA) followed by the Dunnett *post hoc* test. Statistical significance was designated as (*) *p* < 0.05, (**) *p* < 0.01, (***) *p* < 0.001, and (****) *p* < 0.0001 when compared to the BCCAO/R group. No significant difference (ns).

## Data Availability

The original contributions presented in the study are included in the article/Supplementary Material, further inquiries can be directed to the corresponding authors.
