# Supplementary material for: Neuroprotective effect of Bouvardia ternifolia (Cav.) Schltdl via inhibition of TLR4/NF-κB, caspase-3/Bax/Bcl-2 pathways in ischemia/reperfusion injury in rats
Source: Front Pharmacol. 2024 Sep 23;15:1471542. doi: 10.3389/fphar.2024.1471542 (PMC11456924; doi:10.3389/fphar.2024.1471542)
Supplement: Supplementary file 1 [file DataSheet1.PDF]

Neuroprotective effect of *Bouvardia ternifolia* (Cav.) Schltdl via inhibition of TLR4/NF-κB, Caspase-3/Bax/Bcl-2 pathways in ischemia\reperfusion injury in rats

Yury Maritza Zapata-Lopera[1,2], Gabriela Trejo-Tapia[2*], Edgar Cano-Europa[4], Aida Araceli Rodríguez-Hernández[3], Plácido Rojas-Franco[4], Maribel Herrera-Ruiz[1] and Enrique Jiménez-Ferrer[1*]

[1] Centro de investigación Biomédica del Sur, Instituto Mexicano del Seguro Social, Xochitepec, 62790, Morelos, México
[2] Centro de Desarrollo de Productos Bióticos, Instituto Politécnico Nacional, Yautepec, 62730, Morelos, México
[3] CONAHCYT - Instituto Politécnico Nacional, Centro de Desarrollo de Productos Bióticos, Yautepec, 62730, Morelos, México
[4] Laboratorio de Metabolismo I, Departamento de Fisiología, Escuela Nacional de Ciencias Biológicas, Instituto Politécnico Nacional, Ciudad de México, 07738, México
*Corresponding: enriqueferrer_mx@yahoo.com; gttapia@ipn.mx

Figure	Marker	Blot	
		Loading order	Sham rats 1-3, BCCAO/R 4-6, Silymarin, 7-9, dichloromethane extract at 75, 150, and 300 mg/kg (10-18)
All figures mentioned above	β-actine	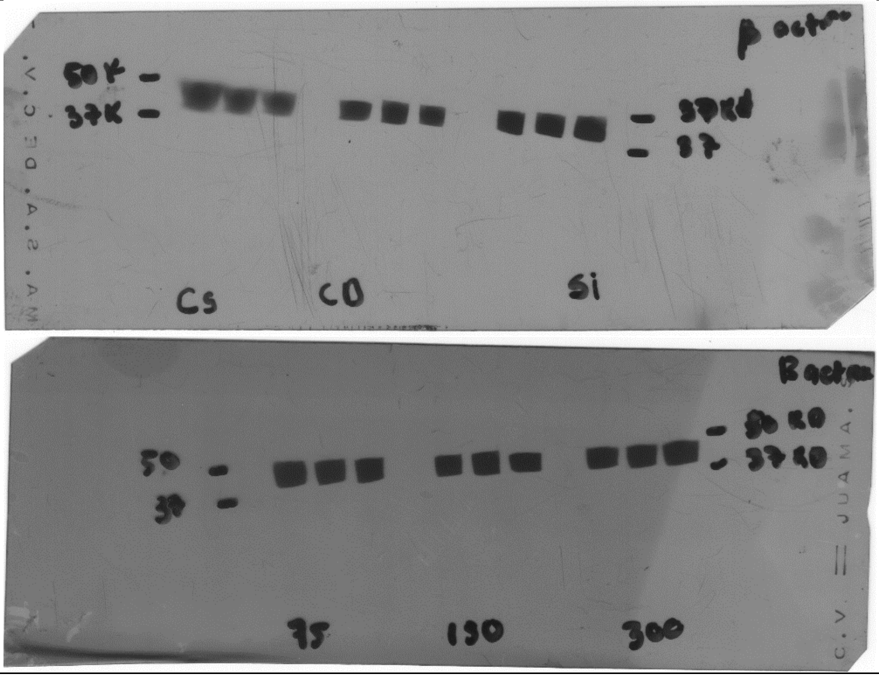	

<table>
<tr><td>Figure 7</td><td>TLR-4</td><td>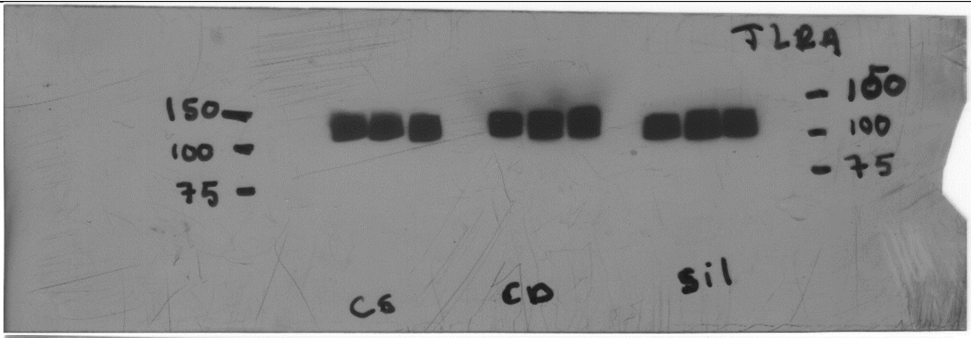

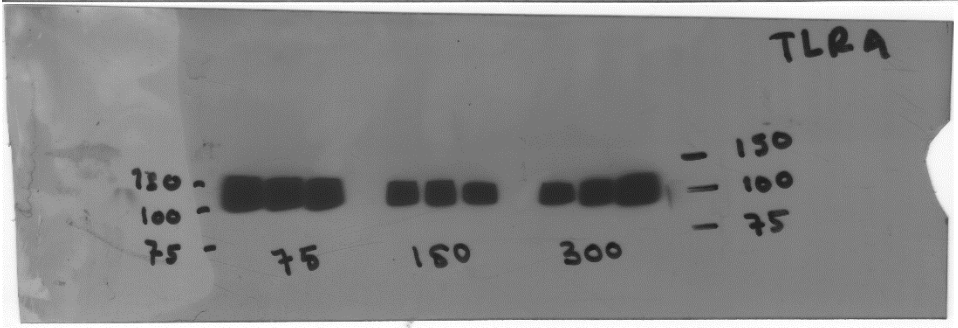
</td></tr>
<tr><td>Figure 4 y 7</td><td>NF-κB</td><td>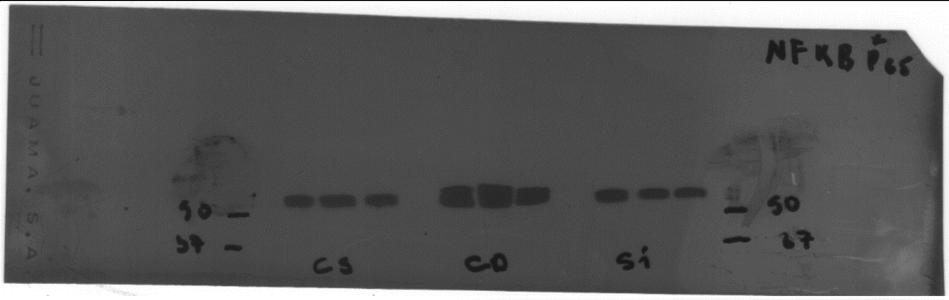

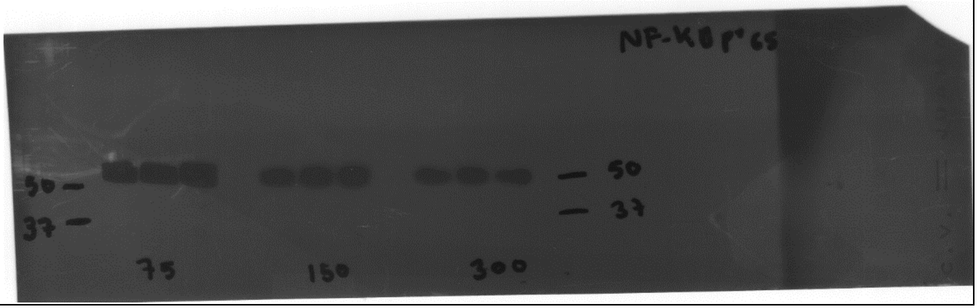
</td></tr>
</table>

Figure 7	nNOS	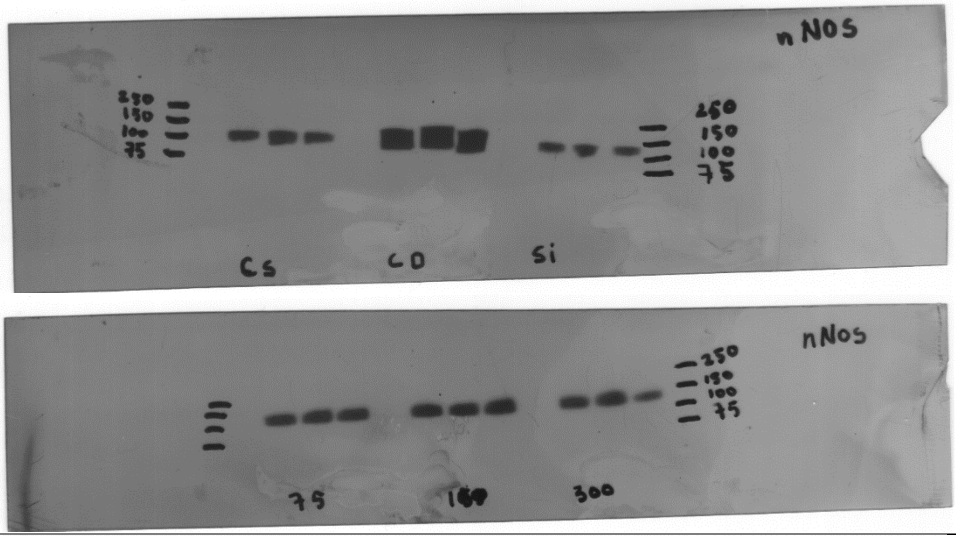
Figure 7	iNOS	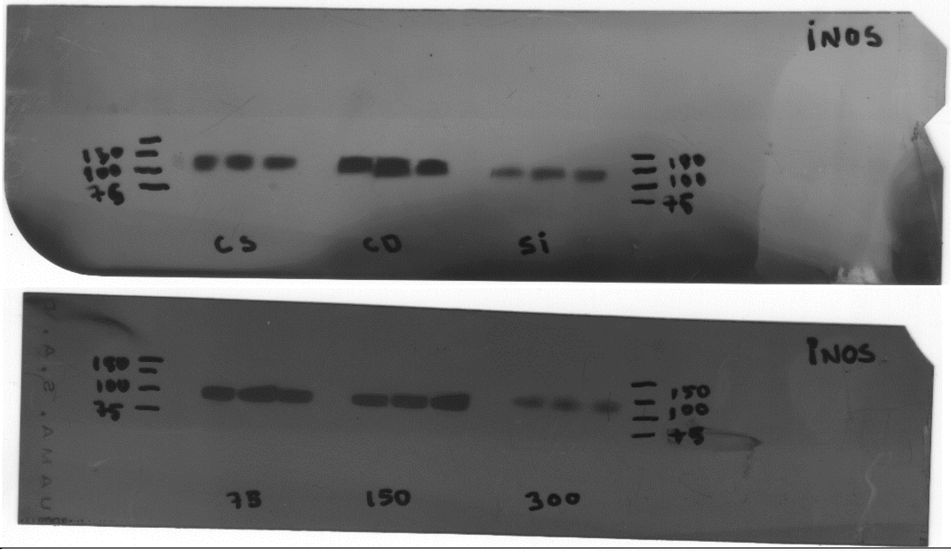
Figure 7	COX-2	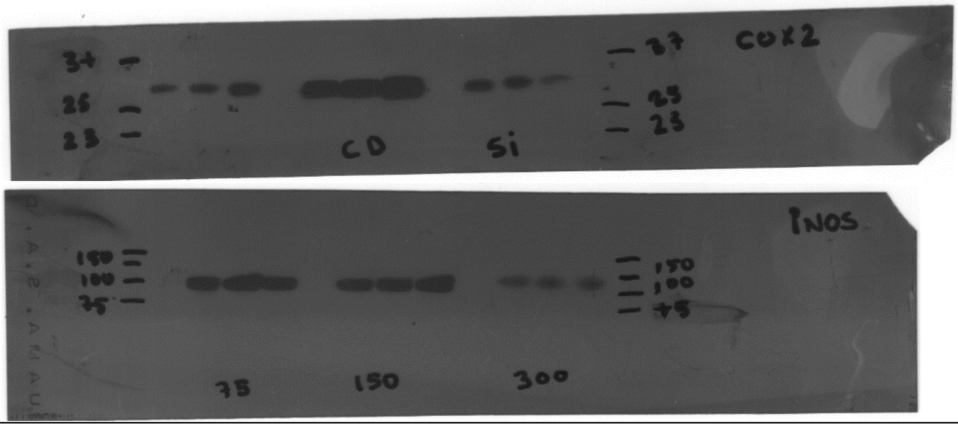

Figure 8	Bcl-2 and Bax	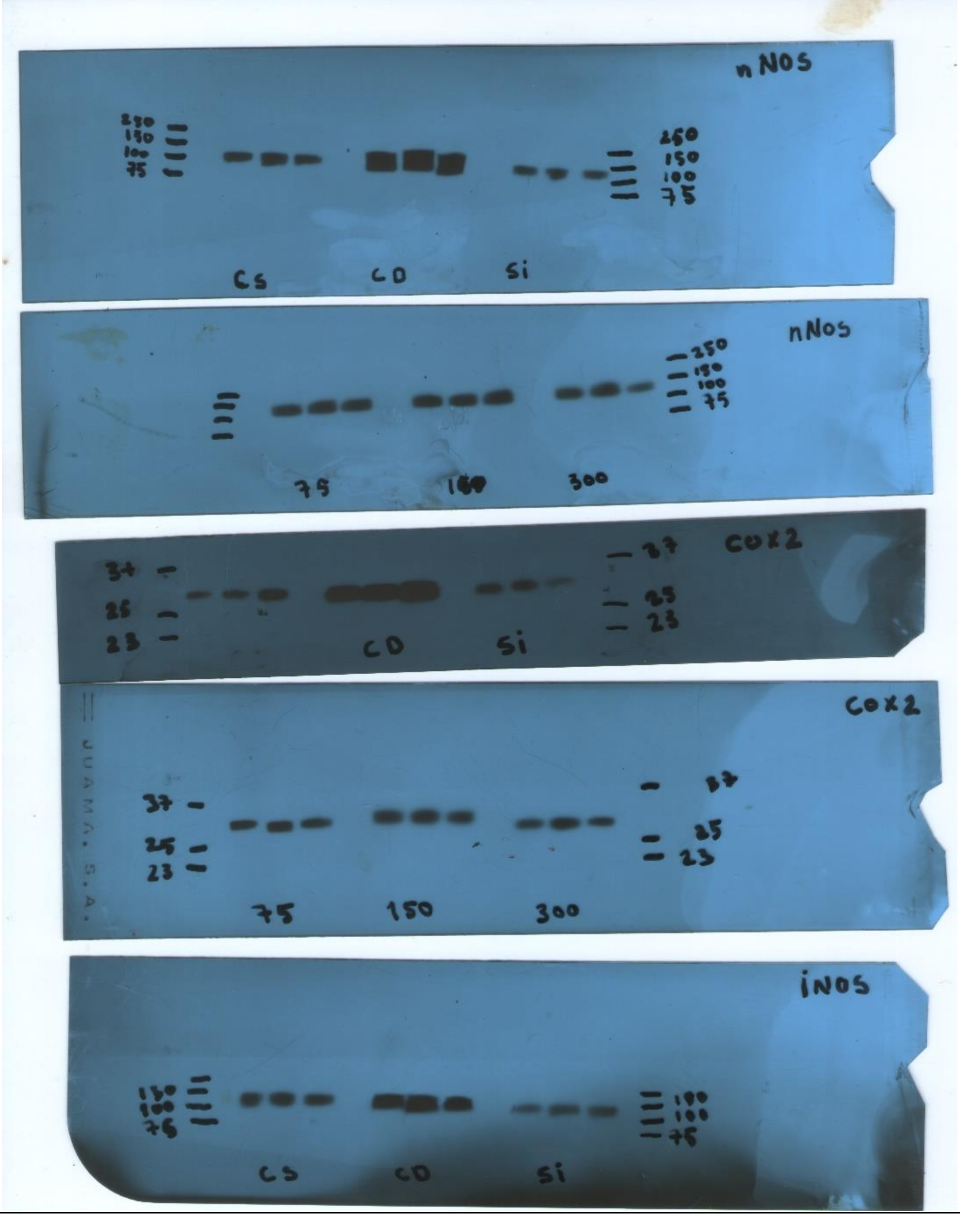
